# A new method for functional analysis of plastid *EMBRYO-DEFECTIVE PPR* genes by efficiently constructing cosuppression lines in *Arabidopsis*

**DOI:** 10.1186/s13007-020-00696-0

**Published:** 2020-11-18

**Authors:** Jingli Chen, Haojie Zhu, Jirong Huang, Weihua Huang

**Affiliations:** Shanghai Key Laboratory of Plant Molecular Sciences, College of Life Sciences, Shanghai Normal University, Shanghai, 200234 China

**Keywords:** *Arabidopsis*, *EMB PPR* genes, Cosuppression, PTGS, RDR6

## Abstract

**Background:**

Pentatricopeptide-repeat proteins (PPRs) characterized by tandem arrays of a degenerate 35-amino-acid repeat (PPR motif) can bind a single strand RNA and regulate organelle gene expression at the post-transcriptional level, including RNA cleavage, splicing, editing and stability etc. PPRs are conserved in all eukaryotes and extremely expanded in higher plants. Many knockout mutants of *PPR* genes are embryonically lethal. These genes are named *EMB PPRs* and functional analysis of them is hindered by the difficulty in obtaining their knockout mutants.

**Results:**

Here, we report a new method for functional analysis of plastid *EMB PPRs* by efficiently constructing their cosuppression lines in *Arabidopsis*. When we overexpressed a mutated full length or truncated coding sequence (CDS) of *EMB PPRs*, such as *EMB2279*, *EMB2654* and *EMB976* (all belong to the P family PPRs) in the wild-type (WT) background, a large portion of T_1_ plants displayed chlorosis phenotypes, which are similar to those of the weak allele mutants, knockdown lines or partially complementary lines. RT-PCR analysis showed that overexpression of the truncated *EMB PPRs* led to significant and specific downregulation of their corresponding endogenous mRNAs. However, when these *EMB PPRs* were overexpressed in the Post transcriptional Gene Silencing (PTGS) deficient mutant, *RNA-dependent RNA polymerase 6* (*rdr6*), none of the T_1_ plants displayed chlorosis phenotypes. These results indicate that the chlorosis phenotype results from post transcriptional silencing of the corresponding endogenous gene (also known as sense cosuppression).

**Conclusions:**

Overexpression of an appropriately truncated *EMB PPR* CDS in WT leads to gene silencing in a RDR6-dependent manner, and this method can be employed to study the unknown function of *EMB PPR* genes. By this method, we showed that EMB976 is required for splicing of chloroplast *clpP1* intron 2 and *ycf3* intron 1.

## Background

It is a common phenomenon that knockout mutants of plant essential genes display embryo or seedling lethal phenotype [1]. In the Arabidopsis genome, there are about 510 *EMBRYO-DEFECTIVE* (*EMB*) genes. Among them, 34 genes belong to the pentatricopeptide-repeat (PPR) gene family [1, 2]. PPR proteins characterized by tandem arrays of a degenerate 35-amino-acid repeat (PPR motif) are conserved in all eukaryotes and extremely expanded in higher plants. It has been known that over 450 PPR proteins are present in *Arabidopsis* and divided into two subfamilies, designated P and PLS, according to the characteristics of the PPR motifs [3]. Most PPRs are involved in regulation of organelle gene expression, including RNA cleavage, splicing, editing and stability, via directly binding to their RNA targets [4]. A set of EMB PPRs have been reported to be required in RNA processing of the chloroplast housekeeping genes. For example, EMB2261 is specifically involved in chloroplast *rps14* mRNA editing [5]. EMB2750 (PPR2) is specifically involved in chloroplast 23S rRNA processing [6]. EMB3140 (PDM3) is specifically involved in chloroplast *trnA* and *clpP1* intron1 splicing [7]. EMB2279/SOT5 is specifically involved in chloroplast *rpl2* and *trnK* intron splicing [8]. EMB2654 and atPPR4 are coordinately and specifically involved in trans-splicing of plastid *rps12* intron1 [9, 10]. And more recently EMB976/PDM4 is reported to be specifically involved in chloroplast group II intron splicing including *clpP1* and *ycf3* [11]. Since the targets of these PPRs are usually housekeeping genes in chloroplasts, it is understandable that knockout mutants of these *PPR*s are embryonically lethal. Thus, generation of weak allele mutants, viable knockdown lines or partially complementary lines of *EMB PPRs* is required for their functional analysis. Currently, techniques such as antisense, RNAi, artificial microRNA are employed to make knockdown lines [12, 13]. Complementation of *emb2654* heterozygous lines with a cDNA carrying the WT coding sequences under the control of the seed-specific *ABSCISIC ACID-INSENSITIVE3* (*ABI3*) promoter is employed to generate the partially complementary lines [10]. In addition, CRISPR/CAS9 techniques are also widely used for constructing knockout or knockdown mutants [14, 15]. However, these techniques are sometimes not efficient to get wanted transgenic plants. For example, small interfering RNA (siRNA) and artificial microRNA (amiRNA), which are about 21–22 nucleotide (nt), often target more than one endogenous genes if the gene sequence is very conserved among its gene family [13]. And the knockdown lines generated by RNAi or artificial microRNA sometimes display very mild phenotypes, which is not helpful for phenotypical study. Constructing partially complementary lines is a time-consuming process due to making transgenic plants in heterozygotes at first [10]. CRISPR/CAS9 techniques usually cause off-target effects, and identification of the genome-edited lines is relatively costly [14].

The gene cosuppression often occurs when a homologous coding sequence is overexpressed in plants [16–18]. This phenomenon is controlled by the post transcriptional gene silencing (PTGS) pathway [12], which is initiated by the conversion of single-stranded RNAs (over accumulated aberrant RNAs) into double-stranded RNAs by RNA-dependent RNA polymerase 6 (RDR6). The double strand RNAs are subsequently sliced into 21–22 nt small interfering RNAs (siRNA) by dicer-like 2 (DCL2) or DCL4. siRNAs are loaded into the RNA-induced silencing complex (RISC) to cleave the target RNAs [19, 20]. The PTGS pathway is well conserved among eukaryotes. Based on this pathway, antisense, RNA interfering (RNAi) and artificial microRNA techniques are developed and widely used to construct knockdown lines for a lot of model organisms to facilitate their gene function studies [12].

Here, we reported a method that can efficiently make cosuppression lines via overexpressing an appropriately truncated *PPR* CDS in the WT background. In addition, we dissected the biological function of a PPR protein, EMB976, in Arabidopsis using this method.

## Results

### Overexpression of the mutated *EMB2279/SOT5* and *EMB2654* CDS in WT leads to RDR6-dependent gene silencing

**Fig. 1:**
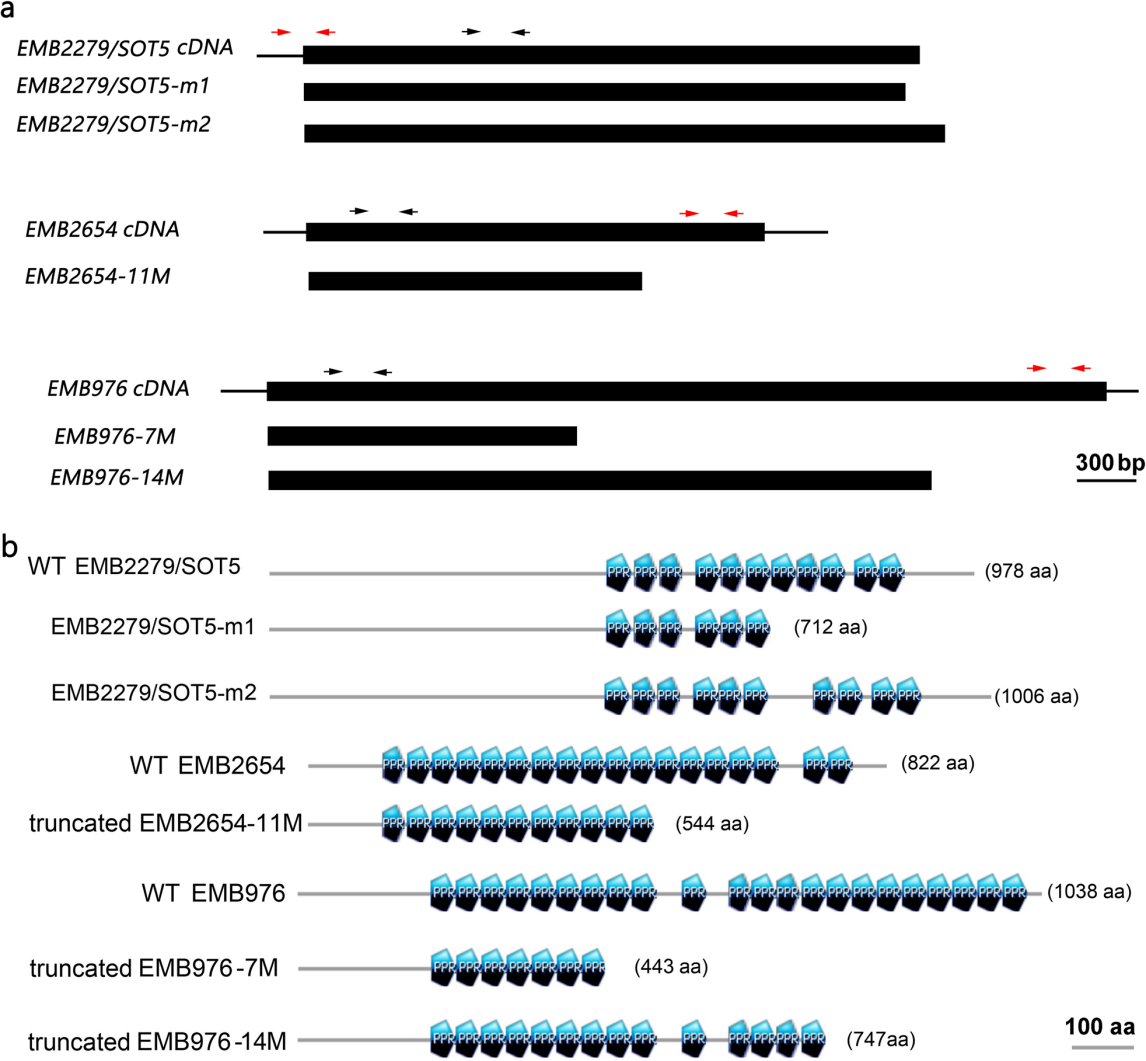
Schematic diagram of the *EMB PPR* CDS constructs made in this study and their coding proteins. **a** Schematic diagram of the *EMB PPR* CDS constructs. The *EMB PPR* CDS is indicated by black box. The UTR regions are indicated by black lines. The black arrows show the position of the primer pair to detect the total genes (including endogenous and transgenes). The red arrows show the position of the primer pair to detect the endogenous genes. Bar = 300 bp. **b** Schematic diagram of the corresponding *EMB PPR* proteins. The protein structures were predicted by PROSITE (https://prosite.expasy.org/). Pentagons mean PPR motifs in each construct. The amino acid (aa) numbers indicate the size of WT or truncated PPR proteins. Bar = 100 aa

**Fig. 2:**
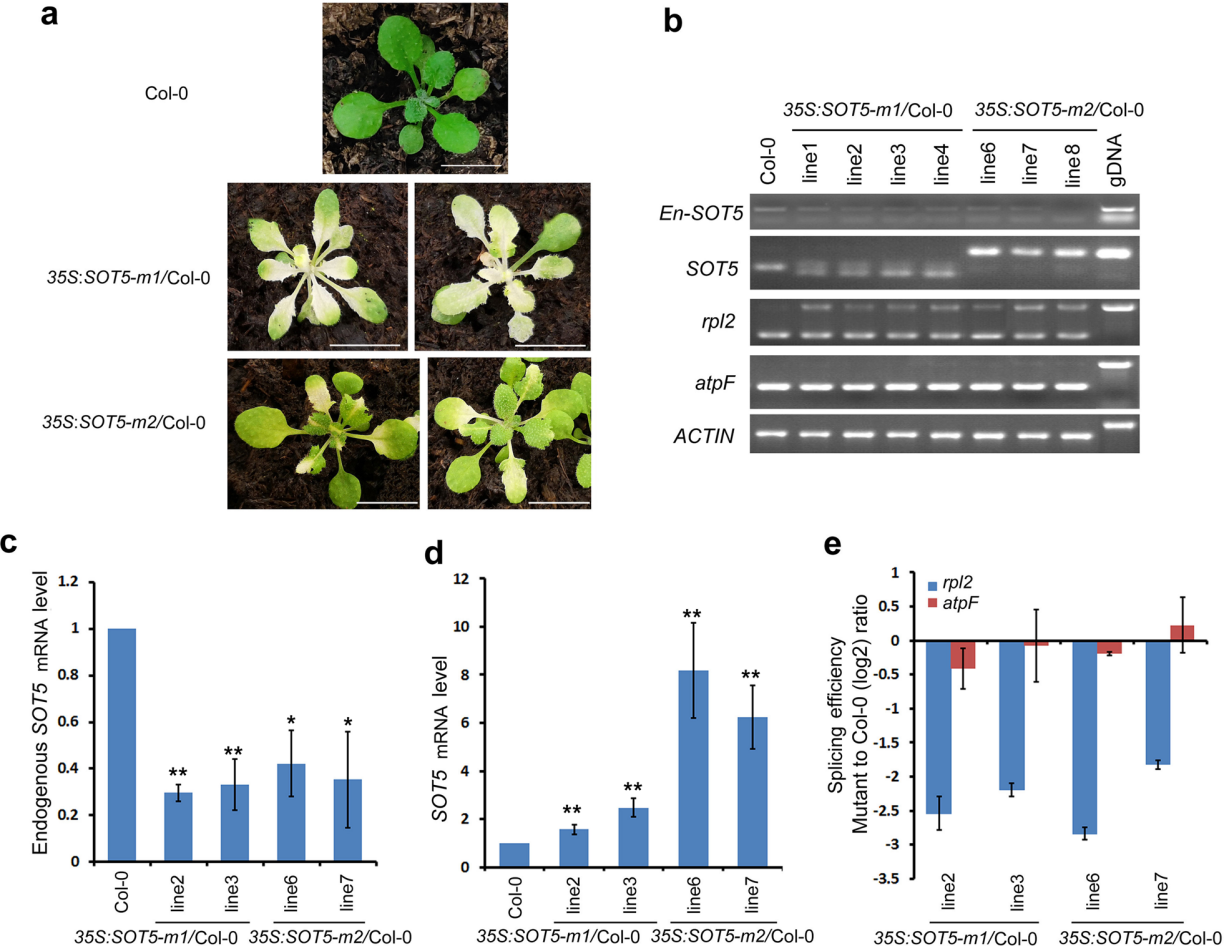
The phenotypes of transgenic plants overexpressing the mutated *SOT5* CDS in WT background. **a** Chlorosis leaf phenotype of transgenic plants overexpressing *SOT5-m1* and* SOT5-m2 *constructs in WT background (bar = 1.5 cm). **b** RT-PCR analysis of expression levels of the endogenous *SOT5*, total *SOT5* (including endogenous *SOT5* and transgene), and two plastid genes in the typical cosuppression lines. **c** RT-qPCR analysis of the endogenous *SOT5* transcript level in the cosuppression lines. **d** RT-qPCR analysis of the total *SOT5* transcript level in the cosuppression lines. **e** Splicing efficiency of plastid *rpl2* and *atpF* (as a control) in the cosuppression lines. For RT-qPCR, the values are means of three technique replicates (bars indicate SD). Asterisks indicate significant differences between wild type (WT) and transgenic plants (Student’s t test, *, P < 0.05 and **, P < 0.01). Two to three T1 transformants were analyzed for RT-PCR and RT-qPCR

We previously reported that a weak allele of *emb2279-2/sot5* mutant exhibits a virescent phenotype, which is caused by a point mutation that significantly reduces splicing efficiency of the seventh intron of *SOT5* and generates two additional mRNA variants [8]. The smallest transcript that lacks 22-base pairs (bp) at the 3′ end of the seventh exon is predicted to produce a truncated SOT5 protein with only 6 PPR motifs, named SOT5-m1 (Fig. 1), whereas the largest transcript that contains the seventh intron is predicted to produce a mutated protein with 10 PPR motifs, named SOT5-m2 (Fig. 1). While the wild type SOT5 encodes a protein with 11 PPR motifs (Fig. 1 and Table 1). To test whether the two predicted proteins are functional or not in plants, we cloned *SOT5-m1* and *SOT5-m2* CDS and transformed them under the control of the cauliflower mosaic virus 35S promoter into WT plants. We obtained 30 and 42 positive T_1_ transformants for *35S:SOT5-m1* and *35S:SOT5-m2* constructs, respectively. Our results showed that 80% of the *35S:SOT5-m1* transgenic lines exhibited severe leaf chlorosis at the early growth stage, and these leaves were gradually turned into pale green later; and 48% of *35S:SOT5-m2* transgenic lines displayed relatively mild leaf chlorosis and virescence (Table 1 and Fig. 2a). This chlorosis and virescent phenotype was similar to that of the *sot5* mutant. Thus, we suspected that the transgenic lines with chlorosis were the cosuppression lines, in which expression of the endogenous *SOT5* was silenced. Then, we analyzed expression levels of *SOT5* in T_1_ transformants with chlorosis. Indeed, RT-PCR and quantitative PCR (qPCR) analyses showed that the level of the endogenous *SOT5* transcripts (*En-SOT5*), detected by the specific primer pair spanning 5′UTR (the red arrows highlighted on *EMB2279/SOT5* cDNA in Fig. 1), was significantly decreased in *35S:SOT5-m1*/Col-0 transgenic line2 and line3 (less than 40% of the WT expression level), whereas the level of total *SOT5* transcripts (including endogenous and exogenous *SOT5* in transgenic lines), detected by the primer pair spanning intron 7 (Fig.2b) or by the primer pair in CDS (black arrows highlighted on *EMB2279/SOT5* cDNA in Fig. 1) was significantly increased (Fig.2c), compared with that in non-transgenic WT plants. Consistently, in *35S:SOT5-m2*/Col-0 transformants, the expression level of endogenous *SOT5* was also significantly decreased (less than 60% of the WT expression level, Fig. 2b, c), while the content of total *SOT5* transcripts was significantly increased (Fig. 2d). Meanwhile, splicing efficiency of the plastid *rpl2* gene, a *SOT5* target, was dramatically reduced in these transformants (Fig. 2b, e). In contrast, splicing efficiency of the plastid *atpF* gene, a non-target gene of *SOT5*, was not altered in all transgenic plants (Fig. 2b, e). These results indicate that overexpression of the mutated *SOT5* CDS in WT leads to suppression of the endogenous gene expression, probably through the post transcriptional gene silencing (PTGS, also known as sense cosuppression) pathway.

**Table 1:** The cosuppression phenotype and frequency of transgenic plants expressing various constructs in WT

Plamid construct^a^	Length of encoding protein (aa)	Number of PPR motifs	Phenotype of cosuppression	Number of total T_1_ transformants	Number of T_1_ transformants with visible chlorosis	Frequency of cosuppression^b^	Severity of chlorosis in cosuppression lines
*SOT5*	978	11	Yellow inflorescence and cauline leaves	35	20	57%	Very mild
*SOT5-m1*	712	6	Albino young leaves at seedling stage	30	24	80%	Strong
*SOT5-m2*	1006	10	Partial albino leaves at seedling stage	42	20	48%	Mild
*EMB2654*	822	18	ND	ND	ND	ND	ND
*EMB2654-11M*	550	11	Chlorosis leaves at seedling stage	47	47	100%	Mild
*EMB976*	1038	22	ND	ND	ND	ND	ND
*EMB976-7M*	443	7	WT-like	13	0	0%	ND
*EMB976-14M*	747	14	Yellow young leaves at seedling stage	8	4	50%	Mild

^a^The pGWB2 plasmid containing the mutated or truncated CDS indicated by the name

^b^Cosuppressed T_1_ tranfromants/total T_1_ transformants

*ND* not determined

**Fig. 3:**
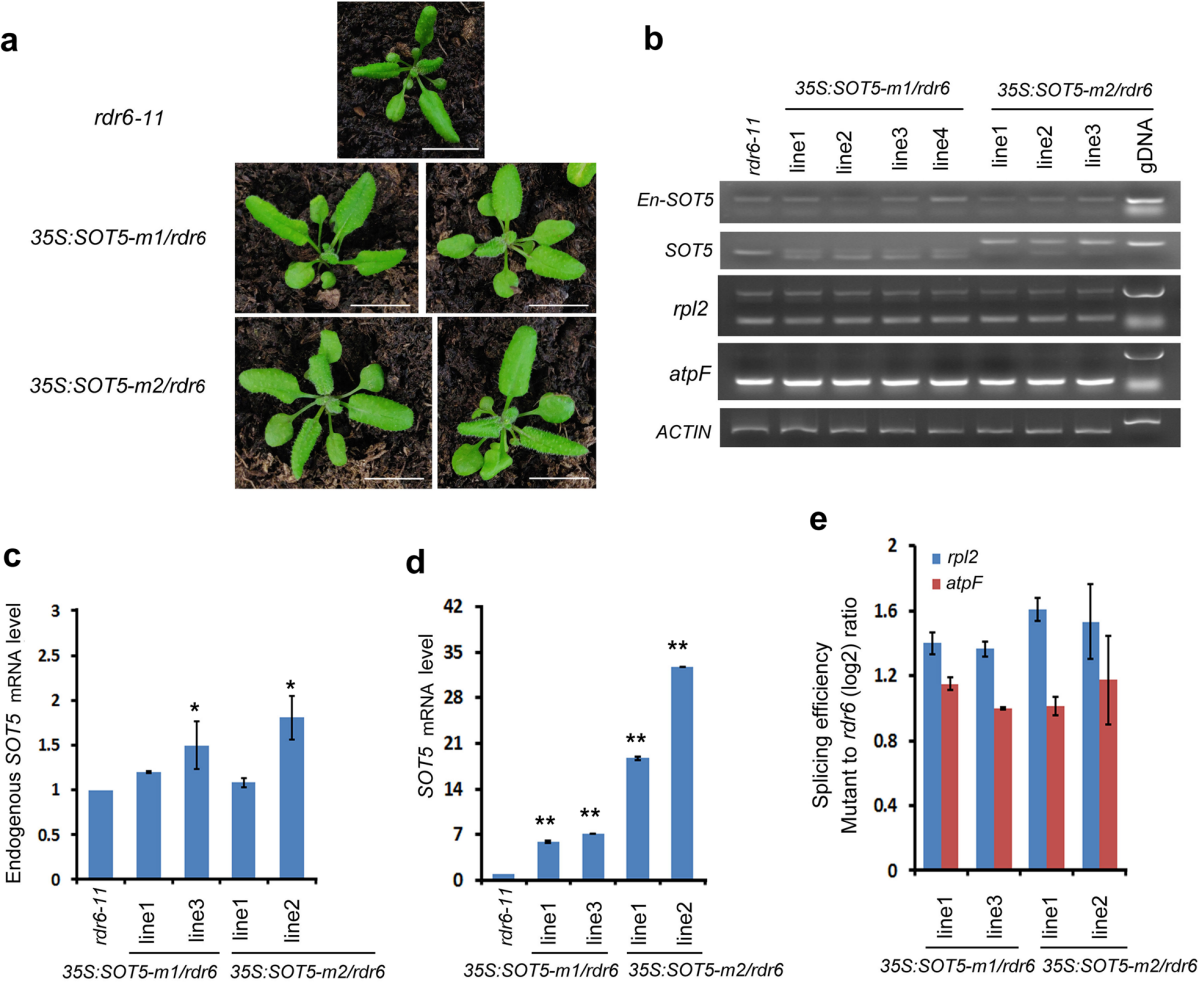
No chlorosis leaf phenotypes appeared when the mutated *SOT5* CDS were overexpressed in *rdr6-11* background. **a** Phenotype of transgenic plants overexpressing *SOT5-m1* and *SOT5-m2* in *rdr6-11* (bar = 1.5 cm). **b** RT-PCR analysis of mRNA levels of endogenous and total *SOT5*, and two plastid genes in transgenic lines. **c** RT-qPCR analysis of the endogenous *SOT5* transcript level in transgenic lines. **d** RT-qPCR analysis of the total *SOT5* expression level in transgenic lines. **e** Splicing efficiency of plastid *rpl2* and *atpF* (as a control) in transgenic lines. For RT-qPCR, the values are means of three technique replicates (bars indicate SD). Asterisks indicate significant differences between wild type (WT) and transgenic plants (Student’s t test, *, P < 0.05 and **, P < 0.01). Two to three T_1_ transformants were analyzed for RT-PCR and RT-qPCR

To confirm this hypothesis, we transformed *35S:SOT5-m1* and *35S:SOT5-m2* constructs into the *rdr6-11* mutant. RDR6 is a key component in the PTGS pathway and the *rdr6-11* mutant presented elongated and curled downward leaves (Fig. 3a) [21, 22]. It has been demonstrated that overexpression of homologous genes in this mutant was not able to trigger the PTGS pathway [21–23]. We obtained twenty-five *35S: SOT5-m1/rdr6* and eighteen *35S: SOT5-m2/rdr6* T1 transformants, and found that none of the transformants exhibited the chlorosis phenotype (Fig. 3a). RT-PCR analysis showed that the endogenous *SOT5* transcript level was not decreased, although the total *SOT5* mRNA level was significantly increased in the transformants (Fig. 3b–d). Consequently, no splicing defect of plastid *rpl2* was detected in these transformants (Fig. 3e). Taken together, our results indicate that overexpression of the mutated *SOT5* CDS in the WT background leads to PTGS in a RDR6-dependent manner.

**Fig. 4:**
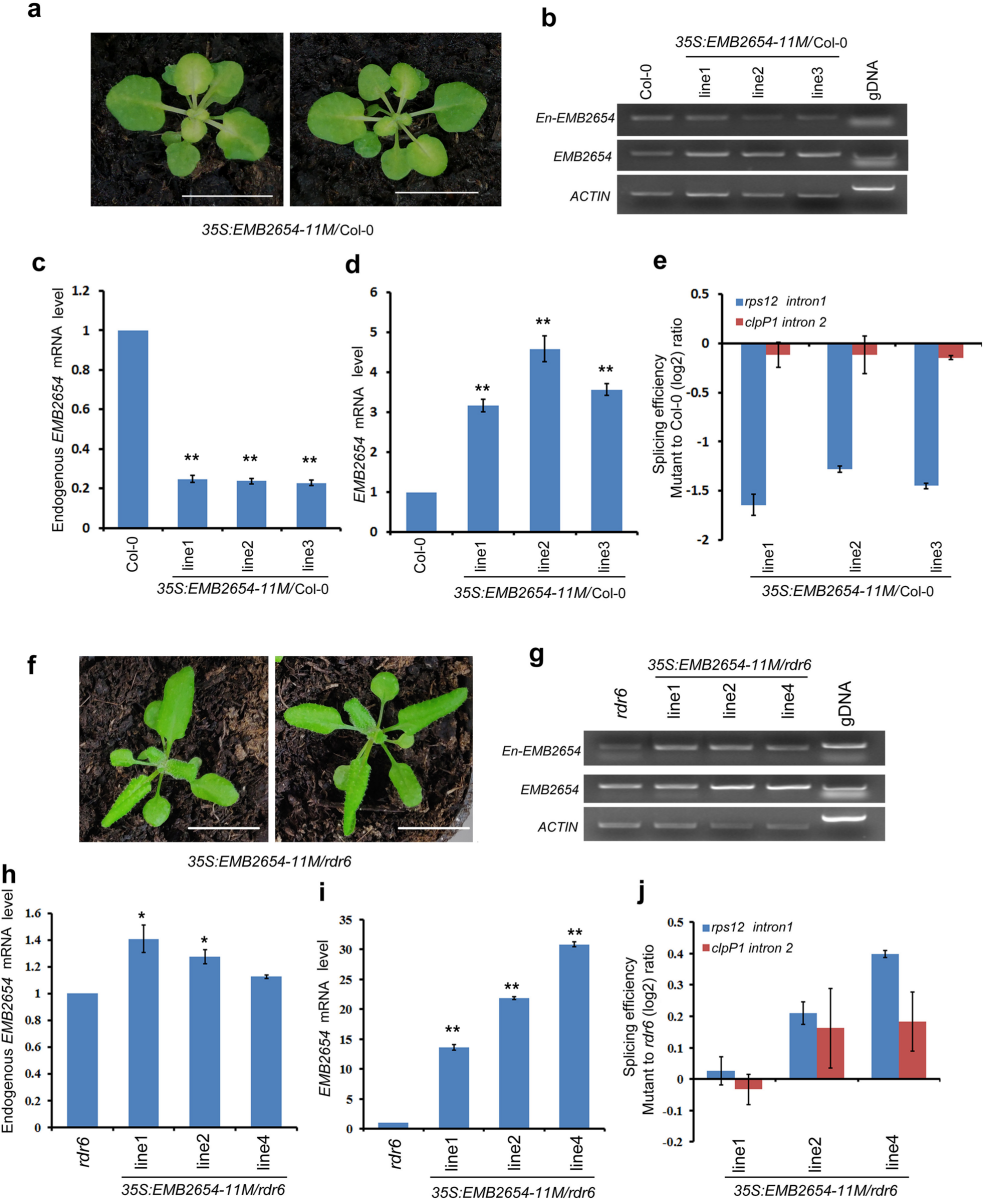
The phenotypes of transgenic plants overexpressing the truncated *EMB2654-11M* are dependent on *RDR6*. **a** Overexpression of the truncated *EMB2654-11M* in WT background leads to chlorosis phenotype (bar = 1.5 cm). **b** RT-PCR analysis of the endogenous and total *EMB2654* transcripts level in the cosuppression lines. **c** RT-qPCR analysis of the endogenous *EMB2654* transcripts level in the cosuppression lines. **d** RT-qPCR analysis of the total *EMB2654* transcripts level in the cosuppression lines. **e** RT-qPCR analysis of the splicing efficiency of plastid *rps12* intron1 in the cosuppression lines. **f** Phenotype of transgenic plants overexpressing *EMB2654-11M* in *rdr6-11* (bar = 1.5 cm). **g** RT-PCR analysis of the endogenous and total *EMB2654* transcripts level in the transformants. **h** RT-qPCR analysis of the endogenous *EMB2654* transcripts level in the transformants. **i** RT-qPCR analysis of the total *EMB2654* transcript level in transgenic lines. **j** Splicing efficiency of plastid *rps12* intron1 in the transformants. For RT-qPCR, the values are means of three technique replicates (bars indicate SD). Asterisks indicate significant differences between wild type (WT) and transgenic plants (Student’s t test, *, P < 0.05 and **, P < 0.01). Two to three T_1_ transformants were analyzed for RT-PCR and qRT-PCR

We then asked whether the above result could be repeated with other *EMB PPR* genes. To address this question, we overexpressed a truncated CDS of *EMB2654* (*EMB2654-11M*) which encodes a truncated protein with only 11 PPR motifs into the WT background (Fig. 1 and Table 1). While the wild type EMB2654 has 18 PPR motifs. It has been reported that EMB2654, a P family PPR protein, was required for trans-splicing of the plastid gene *rps12* intron 1 [10]. Interestingly, all the 47 T_1_ transgenic lines exhibited leaf chlorosis (Table 1 and Fig. 4a), indicating the silence of endogenous *EMB2654*. RT-PCR analysis showed that expression of the endogenous *EMB2654* was down-regulated (about 20% of the WT expression level) while the total mRNA of *EMB2654* was significantly increased in these transformants (Fig. 4b–d). Indeed, the splicing efficiency of *rps12* intron 1 was significantly decreased in the cosuppression lines, while the splicing efficiency of the non-target gene, *clpP1* intron2 was not significantly decreased (Fig. 4e). However, when the truncated *EMB2654-11M* CDS was overexpressed in *rdr6-11*, no leaf chlorosis was observed among 30 transformants (Fig. 4f). RT-PCR results showed that the endogenous *EMB2654* mRNA was not decreased, and the total *EMB2654* mRNA was significantly increased in these transformants (Fig. 4g–i). Consistently, splicing efficiency of the *rps12* intron 1 was not decreased in these transformants (Fig. 4j). Thus, these results confirm that overexpression of the truncated *EMB2654* CDS in the WT background leads to PTGS in a RDR6-dependent manner.

## *Functional analysis of EMB976 *via* its cosuppression lines*

**Fig. 5:**
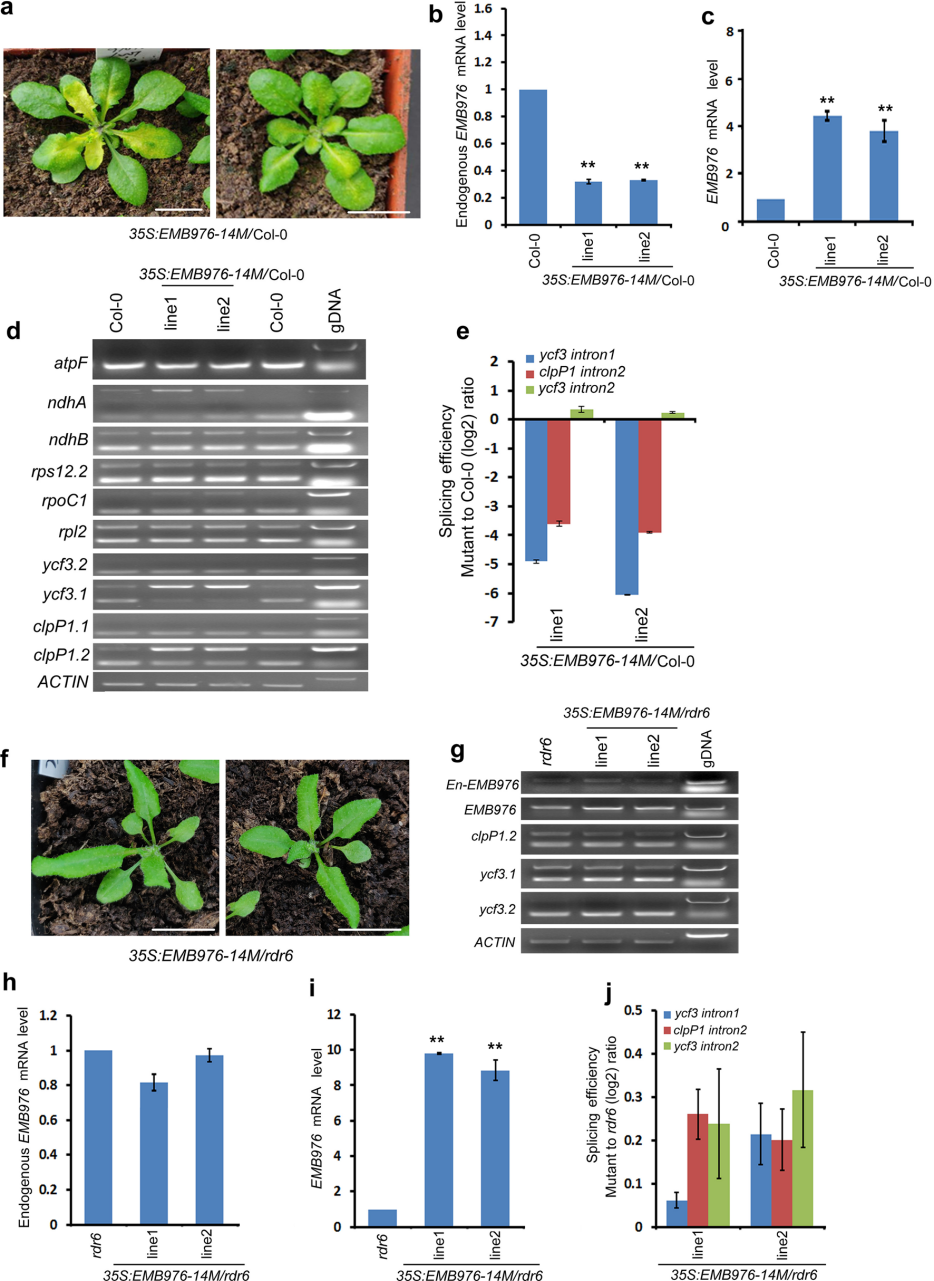
Functional analysis of *EMB976* via overexpressing truncated *EMB976* CDS in WT. **a** Yellow leaf phenotype of T_1_ transgenic plants overexpressing the truncated *EMB976-14M* (bar = 1.5 cm). **b** RT-qPCR analysis of mRNA levels of the endogenous *EMB976* in transgenic lines. **c** RT-qPCR analysis of mRNA levels of total *EMB976* in transgenic lines. **d** RT-PCR analysis of precursor transcripts of chloroplast genes with specific primers. The number after the decimal point indicates the intron of the gene. **e** Splicing efficiency of *clpP1* intron 2 and *ycf3* intron 1 in the cosuppression lines. **f** Phenotype of T_1_ plants overexpressing truncated EMB*976-14M* in *rdr6-11* (bar = 1.5 cm). **g** RT-PCR analysis of mRNA levels of endogenous *EMB976*, total *EMB976*, *clpP1* and *ycf3* precusors in the transformants. **h** RT-qPCR analysis of mRNA levels of endogenous *EMB976* in the transformants. **i** RT-qPCR analysis of mRNA levels of total *EMB976* in the transformants. **j** RT-qPCR analysis of splicing efficiency of plastid *clpP1* intron 2, *ycf3* intron 1 and *ycf3* intron 2 in the transformants. For RT-qPCR, the values are means of three technique replicates (bars indicate SD). Asterisks indicate significant differences between wild type (WT) and transgenic plants (Student’s t test, *, P < 0.05 and **, P < 0.01). Two to three T1 transformants were analyzed for RT-PCR and RT-qPCR

EMB976 is a functionally unknown PPR protein, which belongs to P subfamily containing 22 PPR motifs and is predicted to be localized in chloroplasts. Its knockout mutant has been demonstrated to be embryonically lethal [2]. To study its physiological role in plant growth, we constructed two plasmids named *EMB976-7M* and *EMB976-14M*, which encode the truncated protein with 7 and 14 PPR motifs, respectively (Fig. 1 and Table 1), and transformed them into the WT background. Our results showed that four of eight *35S:EMB976-14M/*Col-0 T_1_ transformants displayed virescent leaves (Fig. 5a and Table 1), whereas all of the thirteen *35S:EMB976-7M//*Col-0 transformants had the same phenotype as WT (Table 1). To confirm whether gene silencing occurred in *35S:EMB976-14M/*Col-0, we checked expression levels of the endogenous *EMB976* in the transformants with the virescent phenotype. As expected, the endogenous *EMB976* was significantly down-regulated (about 40% of the WT expression level) while the total *EMB976* mRNA was significantly increased in these transformants (Fig. 5b, c), indicating that the phenotype of the transgenic lines is caused by the silencing of *EMB976*. Since the P family PPR proteins were often involved in organelle RNA stability and splicing, we further examined the intron splicing of chloroplast genes in these *35S:EMB976-14M/*Col-0 cosuppression lines. Indeed, RT-PCR analysis showed that the precursors of *ndhA*, *clpP1* intron 2 and *ycf3* intron 1 were significantly and specifically accumulated in these lines (Fig. 5d). qPCR analysis showed that the splicing efficiency of *clpP1* intron 2 and *ycf3* intron 1 was significantly and specifically decreased in these lines, compared with that of *ycf3* intron 2 (Fig. 5e), suggesting chloroplast *clpP1* intron 2 and *ycf3* intron 1 were the possible targets of EMB976. However, further experiments are required for verification of the results. Again, no yellow young leaves were observed among the transformants when *EMB976-14M* was overexpressed in *rdr6-11* (Fig. 5f). This result was consistent with that of RT-PCR analysis. In these transformants, the endogenous *EMB976* transcripts was not significantly decreased while the total *EMB976* transcripts was strongly increased (Fig. 5g–i). Consistently, the splicing efficiency of *clpP1* intron 2 and *ycf3* intron 1 was not decreased, compared with that of *ycf3* intron 2 (Fig. 5j). Thus, we conclude that gene silencing triggered by overexpession of truncated *EMB* genes is dependent on RDR6.

## Discussion

### Overexpressing mutated *EMB PPR*s CDS in WT background leads to specific silencing of the endogenous genes

Since it’s hard to get knockout mutants for *EMB* genes, obtaining weak alleles or constructing viable knockdown lines of *EMB* genes is valuable for their functional study. For example, by characterization its weak allele, the PPR protein EMB2279 was disclosed to be specifically involved in chloroplast *trnk* and *rpl2* intron splicing [8]. And by the help of its partial complementation line, EMB2654 was revealed to be specifically involved in trans-splicing of chloroplast *rps12* intron1 [10].

In this study, we found a simple way to make cosuppression lines of *EMB PPR* gene by overexpressing its appropriately truncated CDS fragment in WT background. In the cosuppression lines, the over-accumulated transgene’s transcripts are converted into double-stranded RNAs by RDR6. Then the double strand RNAs are subsequently sliced into 21–22 nt siRNA by dicer-like 2 (DCL2) or DCL4. siRNAs are loaded into the RNA-induced silencing complex (RISC) to cleave the target RNAs [20]. The silenced gene is specifically targeted by its exogenous (transgene) sequence in the cosuppression lines. In our study, the sequence of transgene is long enough (more than or close to 2/3 of WT full length CDS, Table 1), theoretically, the targeting is more specific than that of siRNA or amiRNA. As expected, by RT-qPCR analysis, we found the expression level of *EMB2654* or *EMB976* was not significantly decreased in *35:SOT5-m1*/Col-0 or *35:SOT5-m2*/Col-0 cosuppression lines (Additional file 1: Fig. S1a, b). The expression level of *EMB2654* was not significantly decreased in *35S:EMB976-14M*/Col-0 cosuppression lines and vice versa (Additional file 1: Fig. S1c, d). These results indicate that the targeting is very specific by cosuppression.

Besides the *EMB* genes encoding chloroplasts localized PPR proteins, we also overexpressed an alternative splicing variant of *EMB2784/PRPL4*, which encodes a plastid ribosomal protein L4 into WT background. Compared with the major splicing variant *PRPL4* CDS, the *gPRPL4* CDS retains its unique intron with a premature termination coden (PTC) and leads to a truncated PRPL4 protein. Interestingly, among 9 T_1_ transformants, there were 6 transformants exhibited chlorosis leaves (Additional file 2: Fig. S2a). RT-PCR analysis showed that in these transformants with chlorosis leaves, the endogenous *PRPL4* was significantly decreased (asterisk in Additional file 2: Fig. S2b) and the exogenous *gPRPL4* was increased (arrowhead Additional file 2: Fig. S2b). Meanwhile, RT-qPCR analysis confirmed that *PRPL4* was specifically decreased in the *35S:gPRPL4*/Col-0 cosuppression lines when compared with the expression level of *PRPL28* (*PLASTID RIBOSOMAL PROTEIN L28*, Additional file 2: Fig. S2c, d). These results indicated that overexpression of a mutated CDS of *PRPL4* in WT also leads to cosuppression with a high frequency. Taken together, it seems that this method is universal and robust for constructing knockdown lines for the chloroplast localized EMB proteins. It is likely that dysfunction of the chloroplast-localized proteins often leads to visible chlorosis phenotypes, which makes us easy to know whether the target gene is silenced in transgenic lines. In the future, we will test more genes using this method and try to work out an optimal experimental system to construct cosuppression lines efficiently for *EMB* genes in Arabidopsis.

### The length of truncated PPR CDS is critical for obtaining cosuppression lines with strong mutant phenotype efficiently

We found that cosuppression hardly occurred in the transgenic lines if the length of truncated PPR CDS overexpressed in WT plants was less than half of the full PPR CDS, such as *EMB976-7M* (Table 1). This result is consistent with the report that overexpression of exogenous *Chalcone Synthase* (*Chs*) with a premature translational termination codon (PTC) significantly decreased the frequency of cosuppression, compared with WT *Chs* in Petunia [24]. And Mallory et al. [23] also reported that when the transgene sequence only shared 557 nt of homology with the endogenous AGO1 mRNA, the cosuppression frequency was significantly lower than that using the transgene sequence shares more than 3 kb of homology with the endogenous AGO1 mRNA. The transcripts of transgene with PTC were probably degraded by the nonsense-mediated mRNA decay (NMD) pathway, which can recognize and degrade aberrant transcripts harboring PTC, and thereby prevent the production of truncated proteins which might be deleterious [25]. However, if the PTC occurrs close to the 3′-UTR, the mRNA will escape from the NMD pathway, and thus can accumulate and trigger the RDR6-dependent PTGS pathway [26, 27]. So, theoretically, the full length of CDS will lead to a highest cosuppression frequency. However, we found that when the full length of SOT5 was overexpressed in WT background, only very mild chlorosis (cosuppression) phenotypes were observed at late developmental stage (Additional file 3: Fig. S3), although the cosuppression frequency is high enough (Table 1). In contrast, overexpression of the truncated (more than 2/3 of WT full length CDS, Table 1) or mutated SOT5 CDS in WT led to a high cosuppression frequency and the strong mutant phenotype. It is likely that the absence of endogenous SOT5 protein due to PTGS can be partially compensated by the truncated or mutated SOT5 protein in the cosuppression lines. These results are consistent with the report that the developmental defects of cosuppressed plants were more pronounced in a non-functional AGO1 protein transformants than in a functional AGO1 protein transformants [23]. Thus, we suggest that the appropriate truncation of PPR CDS is critical for constructing cosuppression lines with strong mutant phenotype efficiently.

### EMB976 is required for splicing of chloroplast *clpP1* intron 2 and *ycf3* intron 1

We investigated the role of functionally unknown EMB976 in plant growth by overexpressing the truncated *EMB976-14M* CDS in WT. Our data showed that the *35S: EMB976-14M*/Col-0 cosuppression lines displayed a leaf virescent phenotype and splicing efficiency of *clpP1* intron 2 and *ycf3* intron 1 was significantly deceased in these cosuppression lines. ClpP1 is a core component of ClpP protease complex, which plays critical roles in chloroplast protein homeostasis [28, 29]. Ycf3 is involved in assembly of photosynthetic complex I, which is a key complex in light reaction of photosynthesis [30]. The absence of these critical proteins due to the decreased mature RNAs may explain why the knockout mutant (*emb976*) is embryonically lethal. Interestingly, a recent work showed that EMB976/PDM4 is involved in splicing of group II introns and rRNA processing in chloroplasts [11]. They found the precursors of the chloroplast *ndhA*, *petB*, *ycf3*, *petD* and *clpP1* were present and accumulated in a high level in the *pdm4* knockout mutant but absent in the wild type, which is consistent with our results. The role of EMB976 for group II intron splicing might be independent on its role for rRNA processing. It’s probable that rRNA processing defect is an indirect effect of absence of the chloroplast ClpP protease complex since it was reported that in the weak allele, *clpR4-3* mutant, the plastid rRNA processing was also defective [31].

## Conclusions

Overexpression of an appropriately truncated *EMB PPR* CDS in WT leads to generation cosuppression lines with strong mutant phenotype efficiently, and this method can be employed to study the unknown function of *EMB PPR* genes. By this method, we found that EMB976 is required for intron splicing of plastid *clpP1* and *ycf3*.

## Materials and methods

### Plant materials and growth conditions

The *Arabidopsis* ecotype Columbia-0 (Col-0) was used as WT in this study. The *rdr6-11* mutant used in this study was previously described [22]. Seeds were surface-sterilized by 75% ethanol and stratified at 4 °C for 3 days, and then sown onto half-strength Murashige and Skoog (MS) agar medium with 1% sucrose. Transformants were screened on 1/2 MS agar medium containing 50 μg/ml Kanamycin. About two week old seedlings were transferred into soil. Plants were grown in phytotron under long-day conditions (8 h light/16 h dark) with light intensity (100 μmol photons m^−2^ s^−1^) at 22 °C.

### Plasmid construction and transformation

The mutated *SOT5* CDS (*SOT5-m1* and *SOT5-m2*) was amplified from *sot5* cDNA. The truncated *EMB2654-11M* CDS (1–1635 bp) was amplified from WT cDNA. The truncated *EMB976-7M* CDS (1–1332 bp) and *EMB976-14M* CDS (1–2244 bp) were amplified from WT cDNA, respectively. In order to produce a truncated protein in the transgenic lines, we introduced a stop coden in the reverse primer when we amplifying *EMB2654-11M*,* EMB976-7M* and *EMB976-14M* sequences. The genomic *PRPL4* (*gPRPL4*) was amplified from WT gDNA. The primer sequences were listed in Additional file 4. These amplified sequences were inserted into the pENTR SD/D-TOPO entry vector (Invitrogen). After sequencing, the corrected CDS fragments were recombined into the pGWB2 destination vector as previously described [8]. The destination vectors were transformed into Col-0 or *rdr6-11* mutants using *Agrobacterium* strain GV3101 and the floral-dip method. Transformants were screened on 1/2 MS agar medium containing 50 μg/ml Kanamycin.

### RNA isolation and RT-qPCR

The chlorosis or albino leaves of each cosuppression lines were sampled for RNA extraction. Total RNAs were extracted from leaves of various lines according to the manufacturer’s instructions (Promega Denaturing Solution, Z5651). Downstream DNAse I treatment was performed according to the manufacturer’s instructions (Invitrogen/Gibco DNA-free DNase Treatment & Removal Reagents, AM1906) and reverse transcription (RT) steps were conducted according to the manufacturer’s instructions (Promega Reverse Transcription System, A3500). PCR was performed using the gene-specific primers that are listed in Additional file 4 and according to the manufacturer’s instructions (TaKaRa Taq, R001WZ). Quantitative PCR was carried out using the gene-specific primers that are listed in Additional file 4 according to the manufacturer’s instructions (Takara SYBR Premix Ex Taq, RR420; The Applied Biosystems MicroAmp® Fast Optical 96-Well Reaction Plate-0.1 ml, 4346906; ThermoFisher Scientific QuantStudio™ 3 Real-Time PCR Systems). The data set was normalized using *ACTIN2* as a reference. The method to quantify the transcript level and splicing efficiency of plastid genes was previously described [8, 30]. The expression of endogenous *EMB2279* genes was detected by the primer pair located in 5′UTR (Fig. 1). The total *EMB2279* (including endogenous and transgenes) mRNA was detected by the primer pair located in the coding sequence. The expression of endogenous *EMB2654* or *EMB976* was detected by the primer pair located in the last half of its CDS (Fig. 1). The primers are listed in Additional file 4. In the figures of qPCR, the values are means of three technique replicates (bars indicate SD), asterisks indicate significant differences between wild type (WT) and transgenic plants (Student’s *t* test, *, P < 0.05 and **, P < 0.01). At least two T_1_ transgenic lines were analyzed for each construct.

## Supplementary information

**Additional file 1: Figure S1.** The gene is silenced specifically in each cosuppression line. **a** The expression level of *EMB2654* in *35:SOT5-m1*/Col-0 and *35:SOT5-m2*/Col-0 cosuppression lines. **b** The expression level of *EMB976* in *35:SOT5-m1*/Col-0 and *35:SOT5-m2*/Col-0 cosuppression lines. **c** The expression level of *EMB2654* in *35S:EMB976-14M*/Col-0 cosuppression lines. **d** The expression level of *EMB976* in *35S:EMB2654-11M*/Col-0 cosuppression lines.

**Additional file 2: Figure S2.** The phenotypes of transgenic plants overexpressing the alternative splicing variant (*gPRPL4*) CDS in WT background. **a** Chlorosis leaf phenotype of transgenic plants overexpressing *gPRPL4* in WT background (bar = 2 cm). **b** RT-PCR analysis of expression levels of the *PRPL4* and *PRPL28* in the typical cosuppression lines. The arrowhead indicates the transcripts of transgene *gPRPL4* and asterisk indicates the mature *PRPL4* mRNA. The transgenic line *35S:CFM4*/Col-0 is as a control. **c** RT-qPCR analysis of the mature *PRPL4* mRNA in the typical cosuppression lines. **d** RT-qPCR analysis of expression levels of the *PRPL28* in the typical cosuppression lines. It’s noted that *PRPL28* mRNA was not decreased in the *35S:gPRPL4*/Col-0 cosuppression lines. For RT-qPCR, the values are means of three technique replicates (bars indicate SD). Asterisks indicate significant differences between wild type (WT) and transgenic plants (Student’s t test, *, P < 0.05 and **, P < 0.01).

**Additional file 3: Figure S3.** The phenotypes of transgenic plants overexpressing the full length *SOT5* CDS in the WT background. The chlorosis phenotype of the transgenic lines overexpressing the full length *SOT5* CDS in the WT background appeared at the late developmental stage, such as the chlorosis inflorescence and cauline leaves. Overall, the chlorosis phenotype is not as serious as those cosuppression lines overexpressing the mutated *SOT5* constructs.

**Additional file 4.** Primers used in this study.

## Data Availability

All data generated or analyzed during this study are available in this published article and its additional files.

## Abbreviations

PPR: Pentatricopeptide-repeat proteins
EMB: Embryonic lethal
WT: Wild-type
PTGS: Post transcriptional gene silencing
RDR6: RNA-dependent RNA polymerase 6
DCL2: Dicer-like 2
DCL4: Dicer-like 4
RISC: RNA-induced silencing complex
RNAi: RNA interfering
CDS: Coding sequence
*EMB2279*: *EMBRYO DEFECTIVE2279*
*SOT5*: *SUPPRESSOR OF THF1*
*EMB2654*: *EMBRYO DEFECTIVE2654*
*EMB976*: *EMBRYO DEFECTIVE976*
SOT5-m1: SOT5 mutated protein 1
SOT5-m2: SOT5 mutated protein 2
EMB2654-11M: Truncated EMB2654 protein with 11 PPR motifs
EMB976-7M: Truncated EMB976 protein with 7 PPR motifs
EMB976-14M: Truncated EMB976 protein with 14 PPR motifs
*RPL4*: *PLASTID RIBOSOMAL PROTEIN L4*
*PRPL28*: *PLASTID RIBOSOMAL PROTEIN L28*
*rpl2*: *Plastid ribosomal protein l2*
*rps12*: *Plastid ribosomal protein s12*
bp: Base pairs
nt: Nucleotide
