# Supplementary figures and images for: A new method for functional analysis of plastid EMBRYO-DEFECTIVE PPR genes by efficiently constructing cosuppression lines in Arabidopsis
Source: Plant Methods. 2020 Nov 18;16:154. doi: 10.1186/s13007-020-00696-0 (PMC7673100; doi:10.1186/s13007-020-00696-0)

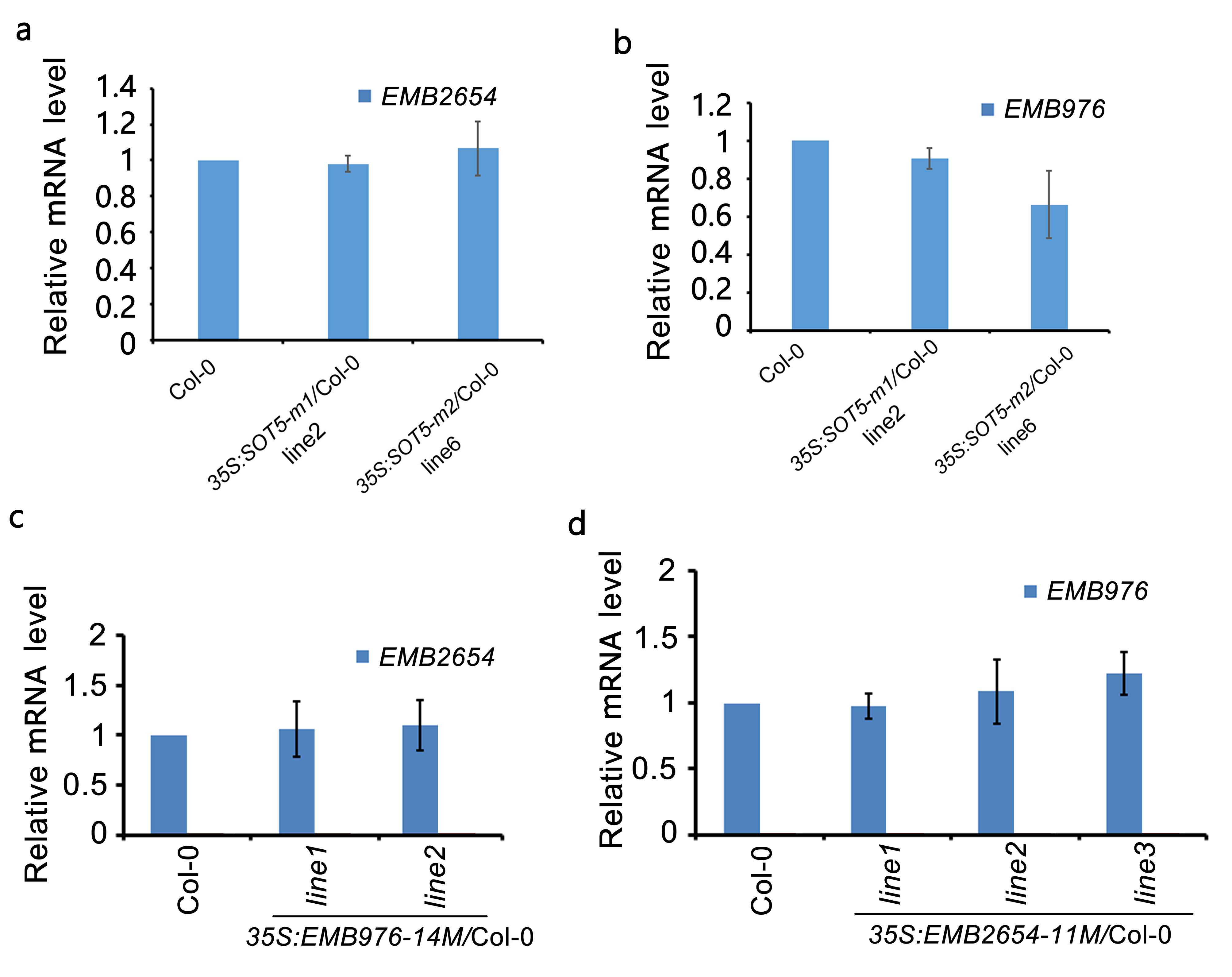

Supplement: Supplementary file 1 — Additional file 1: Figure S1. The gene is silenced specifically in each cosuppression line. a The expression level of EMB2654 in 35:SOT5-m1/Col-0 and 35:SOT5-m2/Col-0 cosuppression lines. b The expression level of EMB976 in 35:SOT5-m1/Col-0 and 35:SOT5-m2/Col-0 cosuppression lines. c The expression level of EMB2654 in 35S:EMB976-14M/Col-0 cosuppression lines. d The expression level of EMB976 in 35S:EMB2654-11M/Col-0 cosuppression lines. [file 13007_2020_696_MOESM1_ESM.tif]

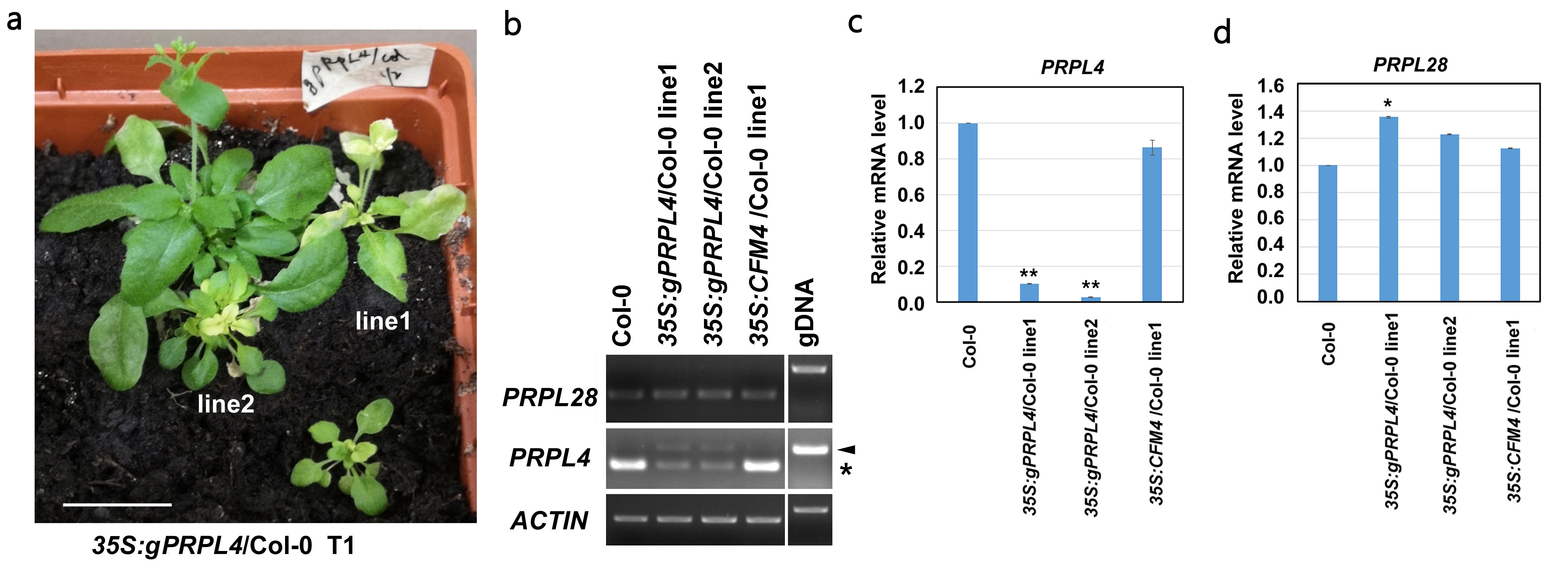

Supplement: Supplementary file 2 — Additional file 2: Figure S2. The phenotypes of transgenic plants overexpressing the alternative splicing variant (gPRPL4) CDS in WT background. a Chlorosis leaf phenotype of transgenic plants overexpressing gPRPL4 in WT background (bar = 2 cm). b RT-PCR analysis of expression levels of the PRPL4 and PRPL28 in the typical cosuppression lines. The arrowhead indicates the transcripts of transgene gPRPL4 and asterisk indicates the mature PRPL4 mRNA. The transgenic line 35S:CFM4/Col-0 is as a control. c RT-qPCR analysis of the mature PRPL4 mRNA in the typical cosuppression lines. d RT-qPCR analysis of expression levels of the PRPL28 in the typical cosuppression lines. It’s noted that PRPL28 mRNA was not decreased in the 35S:gPRPL4/Col-0 cosuppression lines. For RT-qPCR, the values are means of three technique replicates (bars indicate SD). Asterisks indicate significant differences between wild type (WT) and transgenic plants (Student’s t test, *, P < 0.05 and **, P < 0.01). [file 13007_2020_696_MOESM2_ESM.tif]

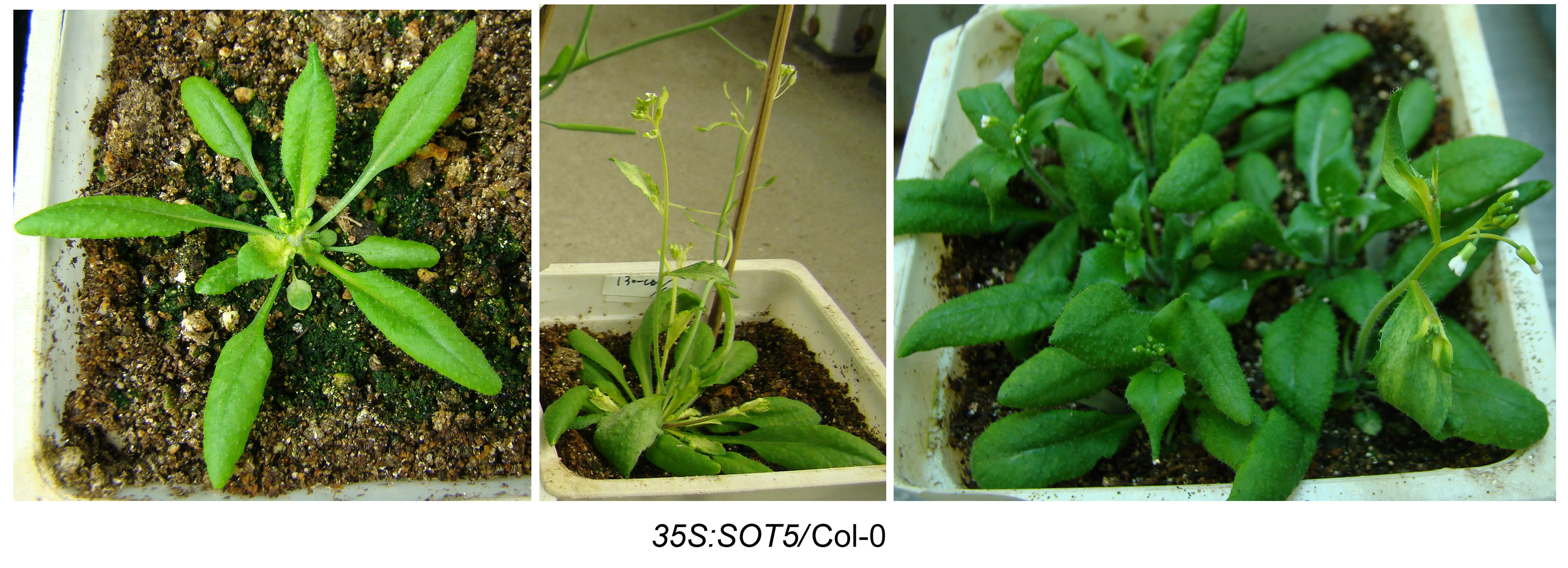

Supplement: Supplementary file 3 — Additional file 3: Figure S3. The phenotypes of transgenic plants overexpressing the full length SOT5 CDS in the WT background. The chlorosis phenotype of the transgenic lines overexpressing the full length SOT5 CDS in the WT background appeared at the late developmental stage, such as the chlorosis inflorescence and cauline leaves. Overall, the chlorosis phenotype is not as serious as those cosuppression lines overexpressing the mutated SOT5 constructs. [file 13007_2020_696_MOESM3_ESM.tif]
